# Controlling the Sense of Embodiment for Virtual Avatar Applications: Methods and Empirical Study

**DOI:** 10.2196/21879

**Published:** 2020-09-22

**Authors:** Chang-Seop Kim, Myeongul Jung, So-Yeon Kim, Kwanguk Kim

**Affiliations:** 1 Department of Computer Science Hanyang University Seoul Republic of Korea; 2 Department of Psychology Duksung Woman’s University Seoul Republic of Korea

**Keywords:** embodiment, virtual avatar, full-body illusion, motion capture, virtual reality

## Abstract

**Background:**

The sense of embodiment (SoE) is the feeling of one’s own body, and research on the SoE extends from the rubber hand illusion to the full-body ownership illusion with a virtual avatar.

**Objective:**

The key to utilizing a virtual avatar is understanding and controlling the SoE, and it can be extended to several medical applications. In this study, we aimed to clarify these aspects by considering the following three subcomponents of SoE: sense of agency, ownership, and self-location.

**Methods:**

We defined a human avatar (HA), point light avatar (PLA), and out-of-body point light avatar (OBPLA) and compared them in three user studies. In study 1, 28 participants were recruited and the three avatar conditions (HA, PLA, and OBPLA) were compared. In study 2, 29 new participants were recruited, and there were two avatar conditions (HA ad PLA) and two motion synchrony conditions (synchrony and asynchrony). In study 3, 29 other participants were recruited, and there were two avatar conditions (PLA and OBPLA) and two motion synchrony conditions (synchrony and asynchrony). Dependent measures included sense of agency, ownership, and self-location; emotional response; presence; and simulator sickness.

**Results:**

The findings of study 1 showed that the three avatar generation methodologies can control the sense of ownership and self-location in a stepwise manner while maintaining a high sense of agency. In studies 2 and 3, we found dependencies among the three subcomponents of SoE and observed that they affected users’ subjective experiences.

**Conclusions:**

Our findings may have implications for boosting the effects of virtual avatar applications in medical areas, by understanding and controlling the SoE with a full-body illusion.

## Introduction

The sense of embodiment (SoE) is the feeling of one’s own body. When we move our bodies in our everyday lives, we feel the SoE unconsciously. The SoE is intimately related to the sense of self and is considered as the starting point of having self-identity [1,2]. The term embodiment has been defined differently in various contexts. From a philosophical perspective, embodiment is considered to be how a person defines and experiences himself/herself [3,4]. In the field of cognitive neuroscience, embodiment is related to how the brain expresses the body [5,6].

Because the SoE is an unconscious process, its alteration or manipulation is difficult. However, Botvinick and Cohen manipulated the SoE using an experiment [7]. With the rubber hand illusion paradigm, the authors demonstrated that participants experienced the SoE on the rubber hand, generating an ownership illusion [7,8]. The rubber hand illusion experiment showed that concurrence of visual and tactile stimulation could generate an embodied experience for a part of one’s body. Recently, researchers have begun to apply virtual reality (VR) techniques to expand the SoE to the whole body. A life-sized virtual avatar for a full-body ownership illusion has been developed [9]. Slater et al showed that participants perceived the ownership illusion from virtual arms [10], and Jun et al demonstrated that a full-body ownership illusion and changes in an avatar’s facial expression meaningfully affected users’ emotions [11].

The effects of the SoE on perceptual and behavioral alterations can be extended to several medical areas. Pioneers have made efforts to apply them in psychological counseling programs [12], and others have used them to enhance cognitive-behavioral therapy for eating disorders [13]. In addition to these applications, systematic desensitized experiences with a virtual avatar could be extended to pain distraction among burn patients, phobias, and posttraumatic stress disorder [14-16]. However, for these purposes, we may need to control the SoE, which has not yet been rigorously examined.

Previous theories have suggested that the SoE can generally be divided into the sense of agency, ownership, and self-location [2]. First, the sense of agency is the subjective feeling that “I am the one who is causing or generating an action” [17]. It includes the subjective experience of action, control, intention, and motor selection, as well as the conscious experience of will [3,18]. Second, the sense of ownership is the feeling that “I am the one who is undergoing the current experience” [17]. The sense of ownership is about the self-attribution of a body that distinguishes it from the sense of agency because it can occur even without a behavior being conducted. Finally, the sense of self-location is the determinate volume in space in which one feels to be located [18]. It is determined by visuospatial perspectives, which are usually egocentric [3], and vestibular signals are also considered to have an important role in self-localization [19].

**Figure 1:**
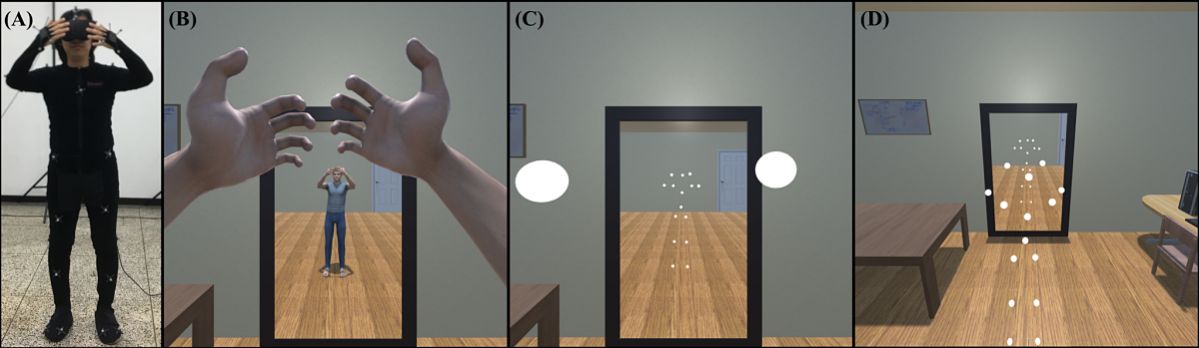
Proposed virtual avatar methodologies. A: real world; B: human avatar condition; C: point light avatar condition; D: out-of-body point light avatar condition.

**Figure 2:**
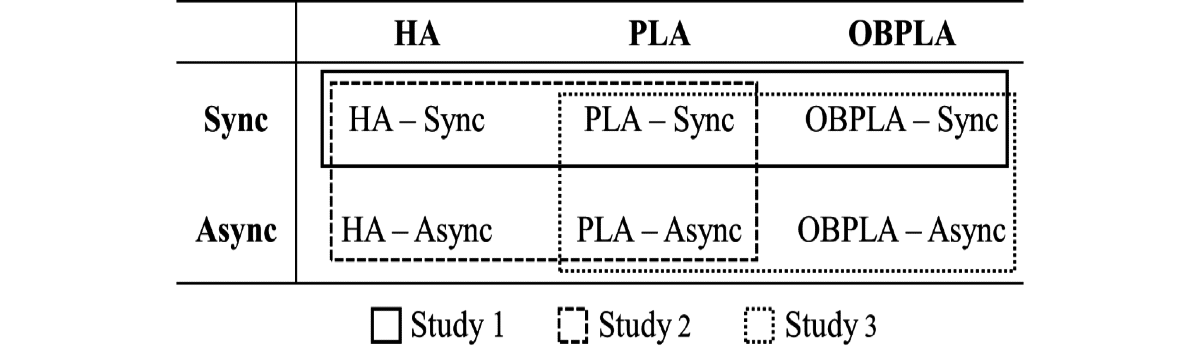
Designs of studies 1, 2, and 3. Async: asynchrony; HA: human avatar; OBPLA: out-of-body point light avatar; PLA: point light avatar; Sync: synchrony.

In this study, we proposed three virtual avatar methods (Figure 1) based on the definition of the three subcomponents of the SoE, as approaches to understand and control the SoE. The first method is human avatar (HA), which is expected to maintain agency, ownership, and self-location at high levels. The HA method uses a human-shaped avatar that moves congruently with the movement of the user and matches the gender and body size of the user [11]. The second method is point light avatar (PLA), which is expected to maintain high levels of agency and self-location but a low level of ownership. The PLA method is based on the point light method proposed by Johansson in his biological motion perception study [20]. The point light is placed at the joint position of the HA to sufficiently reflect the movement of the user but does not have human visual characteristics. Because it does not have human visual characteristics, the PLA is expected to produce a low sense of ownership compared with the HA. However, biological motion is the movement of a living creature and contains information such as behavior, intention, emotion, and personality [21]. Thus, we expect PLA users to be aware of their movement, and their agency will not be lowered. The third method is out-of-body point light avatar (OBPLA), which is expected to maintain a high level of agency and low levels of ownership and self-location. The OBPLA method places the user’s viewpoint behind the PLA so that the user can see the PLA from a third person perspective. Therefore, we expect that the OBPLA will show lower self-location compared with the PLA while maintaining a high level of agency. We defined the HA, PLA, and OBPLA and compared them in three user studies. In study 1, we manipulated the SoE by controlling the presence of the virtual avatar and point of view. In studies 2 and 3, we manipulated the SoE by controlling motion synchrony. The differences among the three studies are illustrated in Figure 2.

## Methods

### Study 1

#### Participants

Twenty-eight participants (50% female participants, 14/28) were recruited for the experiment (mean age 23.89 years, SD 3.08 years). One participant was excluded from the analysis because of having a high risk of mental illness, as assessed by the Symptom Checklist-90-revised (SCL-90-R; mean score 34.07, SD 40.22) [22]. The experimental protocol was approved by the institutional review board of the research site university.

#### Hardware and Software Setups

The virtual environment of the experiment was implemented using Unity3D 2017.3.1f (Unity Technologies). The software was executed on a desktop PC with a Nvidia GeForce GTX 1080 graphics card (NVIDIA). The participants wore a head-mounted display (HMD; Oculus Rift CV1, Oculus VR) with a resolution of 1080×1200 pixels per eye and a refresh rate of 90 Hz.

A motion-capture system (Motive 2.0.2; NaturalPoint) was used with 14 Flex13 cameras (NatrualPoint) and 37 markers on a motion-capture suit. Each Flex13 camera had a resolution of 1280×1024 pixels, a frame rate of 120 frames per second (fps), and a latency of 8.33 ms. The positions of the markers within the tracking area were mapped onto a predefined avatar skeleton using Motive 2.0.2. After generating the avatar skeleton, the values for the skeleton bones with six degrees of freedom were streamed using socket communication at 120 fps with a streaming latency of less than 3 ms.

#### Three Types of Avatars

For this study, the following three avatar conditions were implemented: HA, PLA, and OBPLA.

The HA condition involved moving a human-shaped avatar using a full-body motion capture system. Three Unity3D modules were developed to implement the HA condition. The first module was an avatar animator module, which streamed 21 bones of the skeleton in real time from Motive 2.02 to the avatar. The second module was a size adjuster module, which matched the size of the avatar to the actual participant’s body size using the height, shoulder, waist, and pelvis sizes of the participant measured before the experiment. The gender of each avatar was also matched to that of the participant. The third module was a head rotator module, which received the head rotation information of the HMD and rotated the camera in the virtual space, providing visual information to the participant.

The PLA condition involved moving a PLA using a full-body motion capture system. The PLA was composed of 15 spheres, each with a diameter of 5 cm, according to the study of Troje [21]. The 15 spheres were all white without shadows. In the PLA condition, the avatar animator module sent skeleton data to the PLA, and different from the HA condition, the size adjuster module reflected the height, shoulder, and pelvis sizes. The head rotator module was operated the same as under the HA condition. Therefore, the camera in the virtual space rotated and moved to match the head position of the PLA.

The OBPLA condition utilized the out-of-body experience in the PLA condition, so that the PLA could be seen from the outside. The OBPLA condition was created by moving the camera in the PLA condition backward by 120 cm and upward by 30 cm relative to the global coordinate axis. The participants were able to observe their PLAs from a third person perspective.

#### Virtual Environments

A virtual mirror was placed in the virtual room so that the participants could easily observe their avatar. Participants saw the avatar by both looking directly toward the avatar and by looking at its reflection in the mirror. Several objects, such as computers, a copier, and chairs, were placed in the room for participants to be able to perceive their own body size in relation to these objects.

#### Dependent Measures

As part of a prequestionnaire, personal information, such as gender and age, was obtained, and the SCL-90-R was used. An embodiment questionnaire (EQ) was used to measure the level of the SoE. A part of the EQ developed by Piryankova et al was selected and modified to fit our experiment [23]. The questions in the EQ are explained in detail in Table 1. The EQ used a seven-point Likert scale to measure agency, ownership, and self-location separately. Participants filled out the EQ after experiencing each condition.

**Table 1:** List of the items used in the embodiment questionnaire.

Question statement^a^	Sense aspect
I felt I could move the virtual body.	Agency 1
Sometimes I had the feeling that I had control over the virtual body.	Agency 2
I felt as if the virtual body was my body.	Ownership 1
I experienced the arms of virtual body as parts of myself.	Ownership 2
I experienced the legs of virtual body as parts of myself.	Ownership 3
Sometimes I had the feeling that the virtual body belonged to me.	Ownership 4
I experienced the virtual body as myself.	Ownership 5
I felt as if I was inside the virtual body.	Self-location 1
I had the feeling that I was standing in the same location as the virtual body.	Self-location 2

^a^The scale ranges from 1 (fully disagree) to 7 (fully agree).

#### Procedure

Prior to the experiment, all participants completed consent forms and were instructed on the experimental procedures. All participants also completed a set of prequestionnaires. After the prequestionnaires were completed, participants watched a video clip containing human motion for 5 minutes to prevent participant immobility. Thereafter, participants wore a motion-capture suit and practiced the HA condition for 3 minutes to familiarize themselves with VR and the motion-capture environment. In the main session, participants were asked to move freely in the virtual room under the three conditions. Each condition lasted for 5 minutes and was counterbalanced. After completing each condition, participants removed the HMD and completed the EQ. The purpose of the study was explained to the participants once they had completed all tasks.

### Study 2

Study 2 was designed to address the limitations of Study 1. First, we manipulated the level of SoE by controlling motion synchrony. Second, we investigated the differences in subjective measures achieved by controlling the SoE. Specifically, emotion, presence, and simulator sickness were measured. In study 2, there were two avatar conditions (HA and PLA) and two motion synchrony conditions (synchrony and asynchrony). The differences in experimental design between studies 1 and 2 are illustrated in Figure 2.

#### Participants

Twenty-nine new participants (52% female participants, 15/29) were recruited for study 2. The mean age of the participants was 25.31 years (SD 2.62 years). One of the participants was excluded from the analysis because of the identification of high risk of mental illness according to the SCL-90-R (mean score 39.03, SD 38.41).

#### Conditions

This study implemented the synchrony and asynchrony conditions to control motion synchrony. The synchrony condition was implemented with the avatar animator, size adjuster, and head rotator modules used in study 1. The asynchrony condition used an avatar animation captured in advance instead of delivering the motion capture data to the avatar through the avatar animator module. Although each participant’s movement was not delivered to the avatar, the other conditions were controlled. The size of the avatar was adjusted according to the body size of the participant, and the participant could freely look around even in the asynchrony condition. The avatar conditions (HA and PLA) were the same as those used in study 1.

#### Dependent Measures

In study 2, personal information and the SCL-90-R were used in the same manner as in study 1. The EQ was the same as in study 1. Furthermore, self-assessment manikin (SAM), presence, and simulator sickness questionnaires were completed. The SAM is a nine-point bipolar scale that uses pictures as a measure of participants’ subjective emotional responses to virtual experiences [24]. In this study, arousal and valence were measured as emotional responses. The presence questionnaire (PQ) consists of a seven-point Likert scale and is used to measure presence in VR [25]. The simulator sickness questionnaire (SSQ) is about the degree of simulator sickness and consists of a four-point Likert scale [26].

### Study 3

Study 3 was designed to include the OBPLA condition (OBPLA-sync and OBPLA-async). The PLA and OBPLA were used as avatar conditions, and synchrony and asynchrony were used as motion synchrony conditions. This experiment had a 2×2 within-subject design (Figure 2) considering motion synchrony (sync vs async) and avatars (PLA vs OBPLA).

#### Participants

Twenty-nine participants (48% female participants, 14/29) were recruited for the experiment. The mean age of the participants was 24.14 years (SD 2.74 years). No participants were excluded from the study with the SCL-90-R (mean score 36.86, SD 35.68).

#### Conditions

This study used the same synchrony and asynchrony conditions implemented in study 2 to control motion synchrony. The PLA and OBPLA conditions used in study 1 were used as the avatar conditions.

## Results

### Results of Study 1

**Figure 3:**
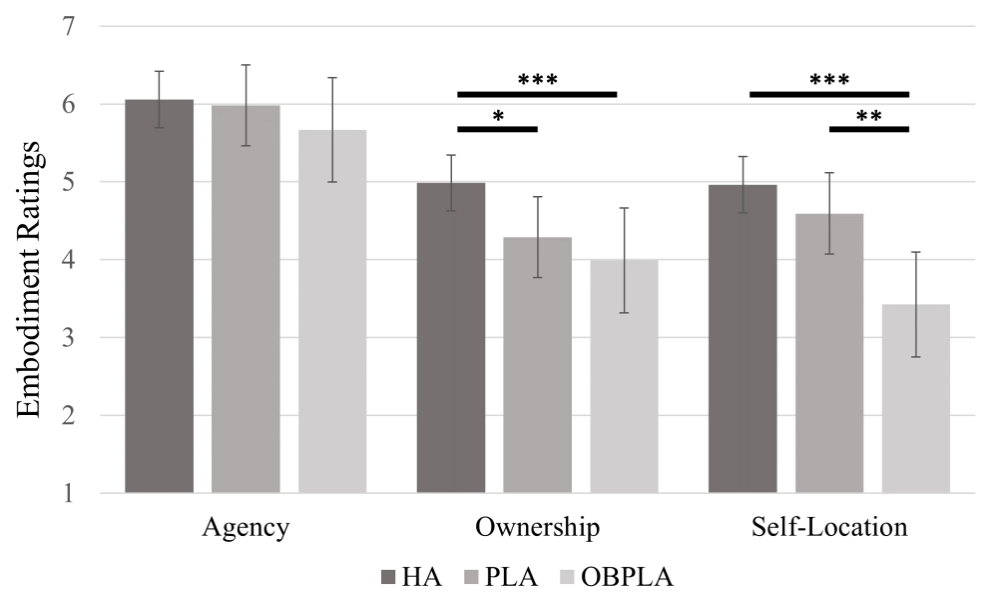
Results of the three subcomponents in the embodiment questionnaire for the three conditions. Error bars are one standard deviation. HA: human avatar; OBPLA: out-of-body point light avatar; PLA: point light avatar. *P<.05, **P<.005, ***P<.001.

We categorized the questions of the EQ in terms of agency, ownership, and self-location. We averaged each category and conducted a repeated measures analysis of variance (ANOVA) to compare the three conditions (HA, PLA, and OBPLA). We performed a *t* test when post-hoc testing was necessary (Figure 3). On comparing the agency scores among the three conditions, there was no statistically significant difference (*P*=.07). On comparing the ownership scores among the three conditions, there was a statistically significant difference (*F*_2,52_=6.239, *P*=.004, η^2^=0.194). In the post-hoc test, the ownership score of the HA was found to be significantly higher than that of the PLA (t_26_=2.245, *P*=.03) and OBPLA (t_26_=3.967, *P*<.001). The ownership scores of the PLA and OBPLA conditions showed no statistically significant difference (*P*=.33). On comparing the self-location scores among the three conditions, there was a statistically significant difference (*F*_2,52_=16.253, *P*<.001, η^2^=0.385). The post-hoc *t* test showed that the self-location scores of the HA and PLA conditions were not significantly different (*P*=.11), but the self-location score of the HA condition was significantly higher than that of the OBPLA condition (t_26_=5.649, *P*<.001). Additionally, the self-location score of the PLA condition was significantly higher than that of the OBPLA condition (t_26_=3.467, *P*=.002).

Study 1 proposed and compared three avatar conditions. In study 1, the level of agency was maintained under all conditions, and the level of ownership could be controlled by comparing the HA and PLA conditions. Moreover, the level of self-location could be controlled by comparing the PLA and OBPLA conditions. These results suggest that the SoE can be controlled through the proposed methods. Although this study suggested that the SoE can be controlled, there are other factors to consider in further studies. First, changes in agency may have an effect. Second, we must consider additional results that can be achieved by controlling the SoE. Previous studies have shown that subjective factors, such as psychological and emotional effects, may play important roles in virtual avatar application [11,27,28], and those could be affected by controlling the agency, ownership, and self-location.

### Results of Study 2

**Figure 4:**
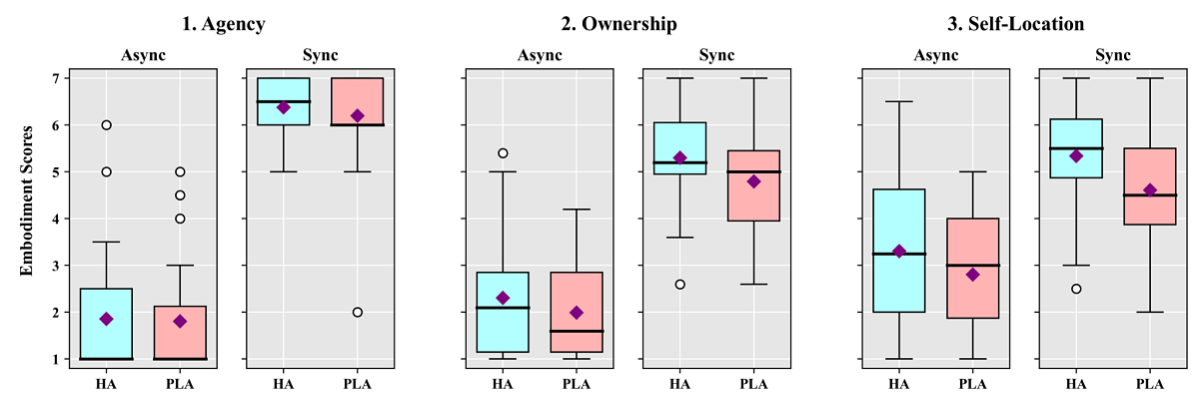
Box-and-whisker diagrams showing the results of the embodiment questionnaire according to the synchrony and avatar conditions in study 2. Square points are means, thick lines are medians, and round points are outliers. Async: asynchrony; HA: human avatar; PLA: point light avatar; Sync: synchrony.

**Figure 5:**
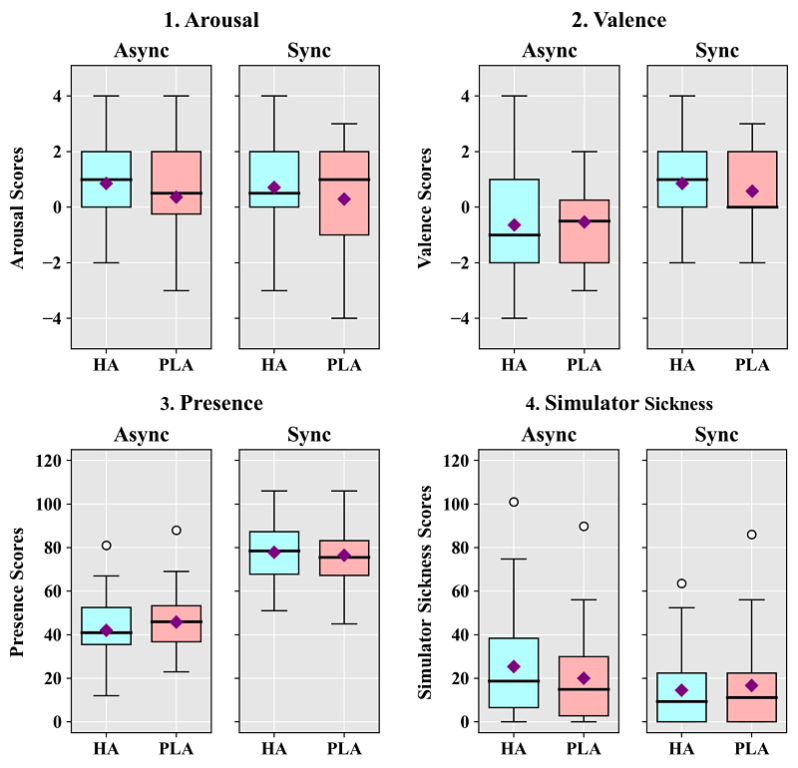
Box-and-whisker diagrams showing the results of the subjective measures according to the synchrony and avatar conditions in study 2. Square points are means, thick lines are medians, and round points are outliers. Async: asynchrony; HA, human avatar; PLA, point light avatar; Sync: asynchrony.

The results of study 2 were analyzed using a 2×2 repeated measures ANOVA with motion synchrony and avatar conditions (Figures 4 and 5). Two factors were analyzed as within-subject factors, and the EQ, SAM, PQ, and SSQ were analyzed. We performed a *t* test when post-hoc testing was necessary.

The agency results showed a significant main effect for motion synchrony (*F*_1,27_=207.328, *P*<.001, η^2^=0.885). There were no significant main effects for avatar (*P*=.36) or between motion synchrony and avatar (*P*=.72). The analysis of ownership showed that there were significant main effects for motion synchrony (*F*_1,27_=102.843, *P*<.001, η^2^=0.792) and avatar (*F*_1,27_=7.293, *P*=.01, η^2^=0.213). However, there was no significant interaction effect between the two factors (*P*=.59). The analysis of self-location showed that there were significant main effects for motion synchrony (*F*_1,27_=37.517, *P*<.001, η^2^=0.582) and avatar (*F*_1,27_=10.318, *P*=.003, η^2^=0.276). However, there was no significant interaction effect between the two factors (*P*=.55).

The SAM scores of each condition were analyzed in terms of the differences from baseline measures. For arousal, the results showed no significant main effect for motion synchrony (*P*=.68), but there was a significant main effect for avatar (*F*_1,27_=5.725, *P*=.02, η^2^=0.175). There was no significant interaction effect between motion synchrony and avatar (*P*=.87). The valence analysis showed that there was a significant main effect for motion synchrony (*F*_1,27_=25.354, *P*<.001, η^2^=0.484). However, there was no significant main effect for avatar (*P*=.61) and no significant interaction effect between the two factors (*P*=.23). The PQ results showed a significant main effect for motion synchrony (*F*_1,27_=85.607, *P*<.001, η^2^=.760), but there was no significant main effect for avatar (*P*=.29). There was a significant interaction effect between motion synchrony and avatar (*F*_1,27_=6.063, *P*=.02, η^2^=0.183). Post-hoc *t* tests showed that PQ scores were not significantly different between the HA and PLA under the synchrony condition (*P*=.43), but the PQ scores under the PLA condition were significantly higher than those under the HA condition in the asynchrony condition (t_27_=−2.869, *P*=.008). The SSQ analysis showed that there was a significant main effect for motion synchrony (*F*_1,27_=4.784, *P*=.04, η^2^=0.151). There was no significant main effect for avatar (*P*=.46) and no significant interaction effect between the two factors (*P*=.12).

### Results of Study 3

**Figure 6:**
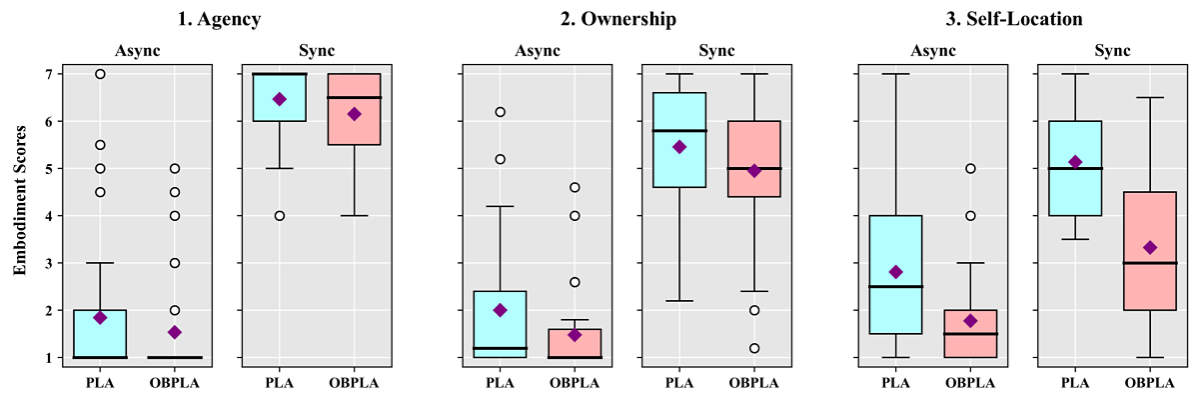
Box-and-whisker diagrams showing the results of the embodiment questionnaire according to the synchrony and avatar conditions in study 3. Square point are means, thick lines are medians, and round points are outliers. Async: asynchrony; OBPLA: out-of-body point light avatar; PLA: point light avatar; Sync: asynchrony.

**Figure 7:**
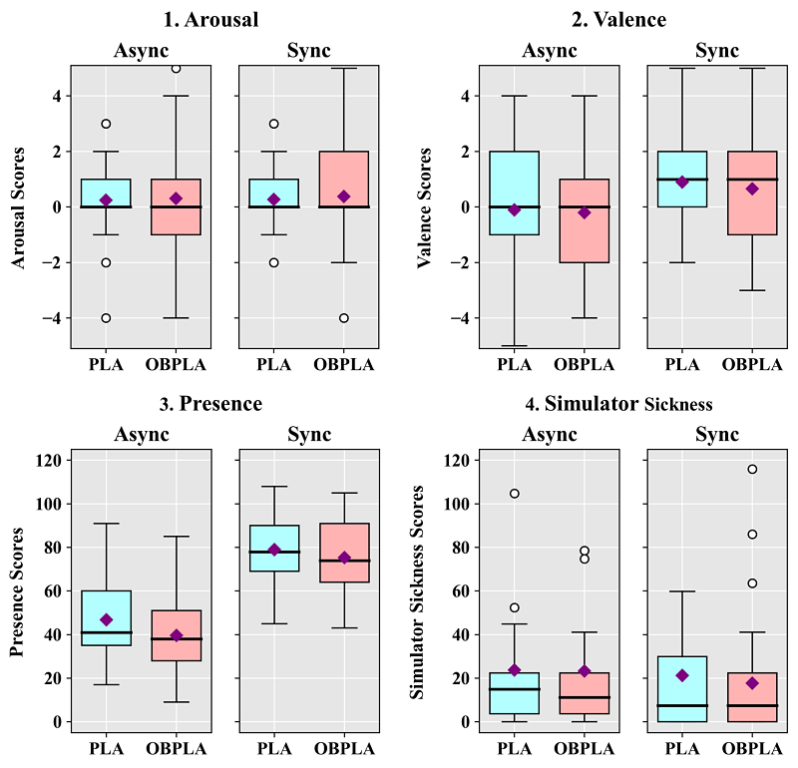
Box-and-whisker diagrams showing the results of the subjective measures according to the synchrony and avatar conditions in study 3. Square points are means, thick lines are medians, and round points are outliers. Async: asynchrony; OBPLA: out-of-body point light avatar; PLA: point light avatar; Sync: synchrony.

The results of study 3 were analyzed using a 2×2 repeated measures ANOVA with the motion synchrony and avatar conditions (Figures 6 and 7).

The agency results showed significant main effects for motion synchrony (*F*_1,28_=218.892, *P*<.001, η^2^=0.887) and avatar (*F*_1,28_=6.610, *P*=.02, η^2^=0.191). However, there was no significant interaction effect between motion synchrony and avatar (*P*=1.00). The analysis of ownership showed significant main effects for motion synchrony (*F*_1,28_=122.502, *P*<.001, η^2^=0.814) and avatar (*F*_1,28_=14.283, *P*<.001, η^2^=0.338). However, there was no significant interaction effect between the two factors (*P*=.97). For self-location, the results showed significant main effects for motion synchrony (*F*_1,28_=40.088, *P*<.001, η^2^=0.589) and avatar (*F*_1,28_=40.180, *P*<.001, η^2^=0.589). There was a significant interaction effect between motion synchrony and avatar (*F*_1,28_=5.156, *P*=.03, η^2^=0.156). In the post-hoc test, the self-location score of the PLA was significantly higher than that of the OBPLA under the synchrony condition (t_28_=7.007, *P*<.001). Under the asynchrony condition, the self-location score of the PLA was also higher than that of the OBPLA (t_28_=3.405, *P*=.002), but the difference was greater under the synchrony condition.

The arousal results showed no significant main effects for motion synchrony (*P*=.75) and avatar (*P*=.56). There was no significant interaction effect between motion synchrony and avatar (*P*=.94). The valence results showed that there was a significant main effect for motion synchrony (*F*_1,28_=20.190, *P*<.001, η^2^=0.419). However, there was no significant main effect for avatar (*P*=.29) and no significant interaction effect between the two factors (*P*=.76). The PQ results showed a significant main effect for motion synchrony (*F*_1,28_=87.779, *P*<.001, η^2^=0.758) and a significant main effect for avatar (*F*_1,28_=6.881, *P*=.01, η^2^=0.197). However, there was no significant interaction effect between motion synchrony and avatar (*P*=.26). The analysis of the SSQ showed that there was a significant main effect for motion synchrony (*F*_1,28_=7.095, *P*=.01, η^2^=0.202). However, there was no significant main effect for avatar (*P*=.46) and no significant interaction effect between the two factors (*P*=.47).

## Discussion

In this research, we proposed virtual avatar methodologies to understand and control the SoE and compared the HA, PLA, and OBPLA conditions using full-body motion capture and VR technologies. Study 1 suggested that it is possible to control the level of SoE from the HA to PLA conditions and from the PLA to OBPLA conditions. In studies 2 and 3, we manipulated motion synchrony and avatar type to determine whether such manipulations affect the SoE, emotional response, presence, and simulator sickness. The results suggested that motion synchrony affects all subtypes of SoE. Regarding avatar type, differences in both ownership and self-location were found between the HA and PLA conditions, indicating positive effects of the HA compared with the PLA for those two SoE variables. Additionally, we found that the PLA increased agency, ownership, and self-location compared with the OBPLA. Presence, emotion, and simulator sickness were affected by motion synchrony and avatar conditions. Our findings suggest that the SoE and subjective experiences can be affected differently with different avatar types in the full-body illusion. We believe that these results can be applied to various fields. For example, the results can be used for systematic desensitization of phobias or pain reduction by controlling the level of SoE. They can also be used to control the effects of emotion and presence associated with using virtual avatars.

In study 1, no relevant differences were found for agency among the three conditions. Despite the absence of human visual characteristics, if full-body motion-capture is maintained, the level of agency is not lowered. Recently, Zopf et al [29] compared virtual fingers and a virtual sphere moving correspondingly to a participant’s hand and found that the moving sphere did not lower the level of agency. This study suggests that the degree of visual stimulation does not affect the sense of agency for not only the hand but also the whole body. Therefore, based on existing research and this study, we suggest that movements induced by one’s intent and predictions determine the sense of agency. Moreover, in the comparison among the avatar conditions, we found that the details of the avatar’s visual characteristics did not affect the sense of agency.

In studies 2 and 3, we investigated the effects of motion synchrony on sense of agency, ownership, and self-location, and the results suggested that motion synchrony greatly affects all SoE subcomponents. Interestingly, the effects of motion synchrony overwhelmed all other effects. Therefore, motion synchrony may need to be controlled if we wish to control the SoE in general. However, the results of study 1 also suggested that the sense of body ownership and self-location can be controlled once motion synchrony is satisfied. Merging the results from studies 1, 2, and 3, we expect that we can use motion synchrony for the general control of the SoE, and we can use the presence of the virtual body and point of view for more detailed control over the SoE. The sense of ownership under the HA condition was higher than that under the PLA and OBPLA conditions. This result is an extension of previous findings that the sense of ownership is induced when the virtual body and physical body are morphologically coincident [7,30,31]. This also suggests that the HA and PLA are appropriate tools for controlling the level of ownership. The point light (PL) method is known to have sufficient information to not only express human behavior but also determine gender [32,33] and recognize emotion [34]. On the other hand, human visual features, such as important interjoint expressions, finger expressions, rotation of some joints, and skin texture are excluded in the PL method. These characteristics of the PL method seem to be suitable for controlling ownership when the PLA is used as a virtual body. Participants experienced a higher sense of self-location under the HA and PLA conditions than under the OBPLA condition. This result supports previous findings that self-location can be controlled through viewpoint changes in low agency situations [35,36]. This study suggested that the control of self-location through changes of perspective is also possible in a high agency situation, such as in a full-body motion-capture system. In study 1, there was no relevant difference in the sense of self-location between the HA and PLA conditions, but in study 2, there was a difference in the sense of self-location according to the avatar’s visual characteristics. Moreover, in study 3, we found that changes in the viewpoint (first vs third person perspective) affected the sense of agency. This suggests the possibility of mutual dependencies among the three subcomponents or the possibility of the context effect of synchrony and asynchrony. However, additional studies are required on this issue.

This study also found that there were significant differences in emotional response, presence, and simulator sickness according to avatar type and motion synchrony. Although the design of this study did not include an emotional evocation context, it was shown that the participants felt different emotional levels depending on the condition. Participants felt higher arousal under the HA condition than under the PLA condition. The HA expresses a human face, but the PLA expresses the face as point light, so the HA contains more facial information than the PLA. Although the avatar under the HA condition had a neutral face, it is possible that the human appearance influenced arousal. The present results also showed an interaction effect between motion synchrony and the avatar’s visual characteristics and a main effect in the avatar’s perspective. It would be beneficial to control presence more precisely by using these combinations of SoE subcomponents.

This study has some limitations. First, the participants in our study were all healthy young college students. It is possible that the characteristics of the participants affected the results of embodiment and subjective responses. Therefore, in future studies, it is necessary to collect data from participants of various ages and health statuses. Second, this study asked participants to move freely and measured arousal and valence as emotions, but pain, anxiety, and fear were not accurately represented. These emotions should be considered for future medical applications. Therefore, in future studies, it is necessary to give specific tasks to participants and check how these methods of controlling the embodiment affect participants in emotional tasks. Third, this study proposed the HA, PLA, and OBPLA to control the external appearance and perspective of the avatar. However, an extension of these methods can be used to control the SoE. For example, we can use lines to express the body or low-polygon human avatars to control the level of visual characteristics. We can also control the point of view, allowing the human avatar to be seen from another third person perspective, or we can see our avatar from the front. Future studies must use additional methods to regulate the SoE and identify the impacts.

In this study, we proposed and compared the HA, PLA, and OBPLA conditions to understand and control the SoE. We also studied how emotional response, presence, and simulator sickness can be affected by controlling the subcomponents of the SoE. The results suggested that the three avatar generation methodologies and two synchrony levels can control the sense of agency, ownership, and self-location, and emotional response, presence, and simulator sickness were differently affected by each methodology. This study may have implications for boosting the effects of virtual avatar applications in several fields of study, such as psychotherapy, entertainment, and education, by controlling the SoE with a full-body illusion.

## Abbreviations

ANOVA: analysis of variance
EQ: embodiment questionnaire
fps: frames per second
HA: human avatar
HMD: head-mounted display
OBPLA: out-of-body point light avatar
PL: point light
PLA: point light avatar
PQ: presence questionnaire
SAM: self-assessment manikin
SCL-90-R: Symptom Checklist-90-revised
SoE: sense of embodiment
SSQ: simulator sickness questionnaire
VR: virtual reality

## References

[1] Cassam Q (1997). Self and World.

[2] Longo MR, Schüür F, Kammers MP, Tsakiris M, Haggard P (2008). What is embodiment? A psychometric approach. Cognition.

[3] Blanke O, Metzinger T (2009). Full-body illusions and minimal phenomenal selfhood. Trends Cogn Sci.

[4] Metzinger T (2008). Empirical perspectives from the self-model theory of subjectivity: a brief summary with examples. Prog Brain Res.

[5] Berlucchi G, Aglioti S (1997). The body in the brain: neural bases of corporeal awareness. Trends Neurosci.

[6] Graziano MS, Botvinick MM, Prinz W, Hommel B (2002). How the brain represents the body: insights from neurophysiology and psychology. Common Mechanisms in Perception and Action: Attention and Performance.

[7] Botvinick M, Cohen J (1998). Rubber hands 'feel' touch that eyes see. Nature.

[8] Armel KC, Ramachandran VS (2003). Projecting sensations to external objects: evidence from skin conductance response. Proc Biol Sci.

[9] Slater M, Spanlang B, Sanchez-Vives MV, Blanke O (2010). First person experience of body transfer in virtual reality. PLoS One.

[10] Slater M, Perez-Marcos D, Ehrsson HH, Sanchez-Vives MV (2009). Inducing illusory ownership of a virtual body. Front Neurosci.

[11] Jun J, Jung M, Kim SY, Kim K (2018). Full-Body Ownership Illusion Can Change Our Emotion. CHI '18: Proceedings of the 2018 CHI Conference on Human Factors in Computing Systems.

[12] Osimo SA, Pizarro R, Spanlang B, Slater M (2015). Conversations between self and self as Sigmund Freud--A virtual body ownership paradigm for self counselling. Sci Rep.

[13] Cesa GL, Manzoni GM, Bacchetta M, Castelnuovo G, Conti S, Gaggioli A, Mantovani F, Molinari E, Cárdenas-López G, Riva G (2013). Virtual reality for enhancing the cognitive behavioral treatment of obesity with binge eating disorder: randomized controlled study with one-year follow-up. J Med Internet Res.

[14] Hoffman HG, Patterson DR, Carrougher GJ (2000). Use of virtual reality for adjunctive treatment of adult burn pain during physical therapy: a controlled study. Clin J Pain.

[15] Reger GM, Gahm GA (2008). Virtual reality exposure therapy for active duty soldiers. J Clin Psychol.

[16] Tardif N, Therrien C, Bouchard S (2019). Re-Examining Psychological Mechanisms Underlying Virtual Reality-Based Exposure for Spider Phobia. Cyberpsychol Behav Soc Netw.

[17] Gallagher S (2000). Philosophical conceptions of the self: implications for cognitive science. Trends in Cognitive Sciences.

[18] Kilteni K, Groten R, Slater M (2012). The Sense of Embodiment in Virtual Reality. Presence: Teleoperators and Virtual Environments.

[19] Lopez C, Halje P, Blanke O (2008). Body ownership and embodiment: vestibular and multisensory mechanisms. Neurophysiol Clin.

[20] Johansson G (1973). Visual perception of biological motion and a model for its analysis. Perception & Psychophysics.

[21] Troje NF (2002). Decomposing biological motion: a framework for analysis and synthesis of human gait patterns. J Vis.

[22] Derogatis LR (1992). SCL-90-R: Administration, scoring & procedures manual-II for the (revised) version and other instruments of the psychopathology rating scale series.

[23] Piryankova IV, Wong HY, Linkenauger SA, Stinson C, Longo MR, Bülthoff HH, Mohler BJ (2014). Owning an overweight or underweight body: distinguishing the physical, experienced and virtual body. PLoS One.

[24] Bradley MM, Lang PJ (1994). Measuring emotion: the Self-Assessment Manikin and the Semantic Differential. J Behav Ther Exp Psychiatry.

[25] Witmer BG, Singer MJ (1998). Measuring Presence in Virtual Environments: A Presence Questionnaire. Presence.

[26] Kennedy RS, Lane NE, Berbaum KS, Lilienthal MG (1993). Simulator Sickness Questionnaire: An Enhanced Method for Quantifying Simulator Sickness. The International Journal of Aviation Psychology.

[27] Kim S, Park H, Jung M, Kim KK (2020). Impact of Body Size Match to an Avatar on the Body Ownership Illusion and User's Subjective Experience. Cyberpsychol Behav Soc Netw.

[28] Yoshie M, Haggard P (2013). Negative emotional outcomes attenuate sense of agency over voluntary actions. Curr Biol.

[29] Zopf R, Polito V, Moore J (2018). Revisiting the link between body and agency: visual movement congruency enhances intentional binding but is not body-specific. Sci Rep.

[30] IJsselsteijn WA, de Kort YA, Haans A (2006). Is This Hand I See Before Me? The Rubber Hand Illusion in Reality, Virtual Reality, and Mixed Reality. Presence: Teleoperators and Virtual Environments.

[31] Sanchez-Vives MV, Spanlang B, Frisoli A, Bergamasco M, Slater M (2010). Virtual hand illusion induced by visuomotor correlations. PLoS One.

[32] Barclay CD, Cutting JE, Kozlowski LT (1978). Temporal and spatial factors in gait perception that influence gender recognition. Percept Psychophys.

[33] Kozlowski LT, Cutting JE (1977). Recognizing the sex of a walker from a dynamic point-light display. Perception & Psychophysics.

[34] Dittrich WH, Troscianko T, Lea SE, Morgan D (1996). Perception of emotion from dynamic point-light displays represented in dance. Perception.

[35] Lenggenhager B, Smith ST, Blanke O (2006). Functional and neural mechanisms of embodiment: importance of the vestibular system and the temporal parietal junction. Rev Neurosci.

[36] Maselli A, Slater M (2013). The building blocks of the full body ownership illusion. Front Hum Neurosci.

